# Individual risk assessment and information technology to optimise screening frequency for diabetic retinopathy by Aspelund et al. (2011) Diabetologia 54:2525–2532

**DOI:** 10.1007/s00417-012-1939-2

**Published:** 2012-03-10

**Authors:** Sarah McGhee, Simon P. Harding, David Wong

**Affiliations:** 1 School of Public Health, LKS Faculty of Medicine, University of Hong Kong, Hong Kong, People’s Republic of China; 2 Department of Eye and Vision Science, Institute of Ageing and Chronic Disease, University of Liverpool, Liverpool, UK; 3 The Eye Institute, LKS Faculty of Medicine, The University of Hong Kong, Hong Kong, People’s Republic of China

The paper by Aspelund et al. [1] reports the potential impact of allocating individuals to different review intervals in screening for sight-threatening diabetic retinopathy (STDR) according to their risk profile. This is an idea which has been around for some time, given that we can identify some of the principal risk factors implicated in determining the risk of STDR over time. Risk engines have also been developed to estimate the risk of stroke and cardiovascular disease [2]. The evaluation of the model developed by Aspelund et al. to determine screening intervals shows that by reducing the number of screens while still detecting the same number of cases of STDR, screening can be more cost-effective. The algorithm has been validated using Danish data collected over a 20-year period, so the model is all the more interesting because of this. However, there are a number of important questions to be considered before widespread implementation.

If we follow the algorithm and the recommendation of their rescreening schedule, the interval between screens could be as long as 60 months. However, the risk status of some patients may change in the interim in terms of blood pressure, blood-sugar control and background diabetic retinopathy (DR). If it is indeed true that risk of complications can be reliably predicted over such a long period, then patients are remarkably true to form. One large cohort study [3] did find that the incidence of retinopathy after 6 years was associated with glycaemia and blood pressure at baseline, although the role of intensive blood-sugar control in prevention of retinopathy is still debated [4].

A population-based study from Norwich, UK which investigated patients managed in general practice between 1990 and 2006 [5] reported that screening intervals of 18–24 months compared to 12–18 months were not associated with a higher risk of development of STDR. However, there was a 60% increase in likelihood of STDR being detected if the screen interval was more than 2 years. Both Iceland and Sweden use extended screening intervals for people at low risk, but these countries have relatively small populations, high levels of funding, and compliance with care. Elsewhere, primary care may not be so reliable, or even absent, and systemic risk factors less well-controlled. In applying the algorithm to another country, we may probably need to take account of not just attendance rates but also different baselines for risk factors and incidence of STDR.

The English National Screening Programme for DR has been reluctant to change to a variable rescreening schedule for a number of practical reasons. The rate of non-attendance for screening can be as high as 30%. Local health providers are responsible for keeping track of the patients, and a longer screening interval adds to the difficulty. Patients may move or default. If any patient comes to harm and develops complications because the system failed to remind them in a repeated and timely fashion, there may be medico-legal consequences. This could be resolved by ensuring a fail-safe follow-up system, although the procedures of this may vary between countries. While having an eye examination at the same time every year might help in adhering to the schedule, more frequent screening such as every 6 months could be seen as an unnecessary inconvenience. The effectiveness of a variable schedule for DR within a centralised screening system requires careful assessment.

The generalisability of the model and longer screening intervals therefore remains unclear. A new screening programme might adopt the concept of risk stratification but modify the details. For example, in the case of Hong Kong, we have decided to use the published algorithm rather than a default annual recall, and we stratify patients' risk, inviting some to come at 6 months but with no assigned interval longer than 2 years. In this way, we may achieve a good compromise, i.e., for those with high risk, we are more likely to detect progression and provide intervention, while seeing everyone within 2 years means that all patients know they have to be seen again, making it possibly less likely that they will fall out of the system. However, reducing the length of the screening intervals for lower risk cases will involve more screening episodes, and less than the 59% saving that adopting the full algorithm might achieve.

In a developing country such as China, where health care provision is non-homogenous and the wealth gap large, a variation of the algorithm may be even more beneficial if it can be implemented safely. For example, screening for DR once every 3–5 years, referring all STDR to an ophthalmologist and, at the same time, referring all high-risk patients without STDR to community clinics for better management of blood pressure and glycaemic control might even achieve more than reduction of blindness, if these important cardiovascular risk factors are reduced. While screening on an annual basis is likely to be impossible in such circumstances, a variable interval with a longer time between screens might detect a sufficient proportion of the STDR cases in a timely manner, and also achieve reduction in overall risk in a substantial proportion of the non-STDR cases.

The risk algorithm as applied by Aspelund et al. potentially achieves savings in costs of screening by shifting the risk of STDR away from those at higher risk but onto those at lower risk, so that the risk of the different groups becomes more similar. This should focus us on equity and the need to ensure that every other means of reducing the risk of the higher risk cases, such as better risk factor control, is being undertaken. It also focuses us on the protocols for ensuring compliance — to more frequent screening in the case of the higher risk groups and longer screening intervals in low risk cases. It reminds us that, especially in countries with a mixed private/public healthcare system, a screening programme is unlikely to achieve full population coverage, and many of those missed will be the higher risk cases. It raises the question of what level of risk of STDR is acceptable in both developed and developing economies.

As we wrestle with the rapid increase in diabetes and the consequent rising prevalence of DR, innovative solutions such as that proposed in the Apselund model merit close study. Real-life evidence and results of further trials will be essential to underpin screening practice, especially for younger patients and those at higher risk, and this evidence needs to be developed for the implementation in widely ranging health care models throughout the world. There are still many questions to answer.

## References

[1] Aspelund T, Thornórisdóttir O, Olafsdottir E, Gudmundsdottir A, Einarsdóttir AB, Mehlsen J, Einarsson S, Pálsson O, Einarsson G, Bek T, Stefánsson E (2011). Individual risk assessment and information technology to optimise screening frequency for diabetic retinopathy. Diabetologia.

[2] Adler AI (2008). UKPDS — modelling of cardiovascular risk assessment and lifetime simulation of outcomes. Diabet Med.

[3] Stratton IM, Kohner EM, Aldington SJ, Turner RC, Holman RR, Manley SE, Matthews DR, for the UKPDS Group (2001). UKPDS 50: Risk factors for incidence and progression of retinopathy in Type II diabetes over 6 years from diagnosis. Diabetologia.

[4] Hemmingsen B, Lund SS, Gluud C, Vaag A, Almdal T, Hemmingsen C, Wetterslev J (2011). Intensive glycaemic control for patients with type 2 diabetes: systematic review with meta-analysis and trial sequential analysis of randomised clinical trials. BMJ.

[5] Misra A, Bachmann MO, Greenwood RH, Jenkins C, Shaw A, Barakat O, Flatman M, Jones CD (2009). Trends in yield and effects of screening intervals during 17 years of a large UK community-based diabetic retinopathy screening programme. Diabet Med.

